# Draft Genome Sequences of *Vibrio* sp. Strains Isolated from Tetrodotoxin-Bearing Scavenging Gastropod

**DOI:** 10.1128/genomeA.00623-14

**Published:** 2014-06-19

**Authors:** Toshiaki Kudo, Ayumi Kawauchi, Tomomi Nakahara, Xiaochi Zhang, Shigeto Taniyama, Tomohiro Takatani, Osamu Arakawa, Kenshiro Oshima, Wataru Suda, Keiko Kitamura, Toshiya Iida, Takao Iino, Tetsushi Inoue, Yuichi Hongoh, Masahira Hattori, Moriya Ohkuma

**Affiliations:** a Graduate School of Fisheries Science and Environmental Studies, Nagasaki University, Nagasaki, Japan; b Graduate School of Science and Technology, Nagasaki University, Nagasaki, Japan; c Center for Omics and Bioinformatics, Graduate School of Frontier Sciences, the University of Tokyo, Kashiwa, Japan; d Japan Collection of Microorganisms/Microbe Division, RIKEN BioResource Center, Tsukuba, Japan; e Department of Biological Sciences, Graduate School of Bioscience and Biotechnology, Tokyo Institute of Technology, Tokyo, Japan

## Abstract

*Vibrio* sp. strains JCM 18905 and JCM 19053 were isolated from a tetrodotoxin (TTX)-bearing scavenging gastropod, and *Vibrio* sp. strain JCM 18904 was isolated from a sea cucumber. All these are closely related to *Vibrio alginolyticus*. Their comparative genome information is useful for studies of TTX production in bacteria.

## GENOME ANNOUNCEMENT

*Vibrio alginolyticus* is a Gram-negative marine bacterium involved in tetrodotoxin (TTX) production (1). It has been elucidated that TTX accumulation in pufferfish is caused by several steps of the food chain, starting with marine bacteria as a primary source of TTX (1). There are several reports of TTX-producing marine bacteria such as *Vibrio alginolyticus*, *Shewanella alga*, *Shewanella putrefaciens*, and *Alteromonas tetraodonis* (1); however, much uncertainty regarding the production mechanisms of TTX in bacteria still remains.

We isolated *Vibrio* sp. strains JCM 18905 (originally designated strain S4) and JCM 19053 (originally designated strain S50) from the TTX-bearing scavenging gastropod *Nassarius* (*Alectrion*) *glans*. This gastropod species is known to bear TTX (2). *Vibrio* sp. strain JCM 18904 (originally designated strain C141) was isolated from the sea cucumber *Holothuria leucospilota* (3). These strains have been deposited at the Japan Collection of Microorganisms (JCM) (http://www.jcm.riken.jp).

The genomes of *Vibrio* sp. strains JCM 18905, JCM 19053, and JCM 18904 were sequenced using an Ion Torrent PGM System. The sequence reads of 558,686 for JCM 18905, 413,036 for JCM 19053, and 557,465 for JCM 18904 were assembled using Newbler version 2.8 (Roche) into 64, 106, and 70 contigs with *N*_50_ lengths of 206,616, 82,571, and 203,785 bp, respectively. These assemblies resulted in draft genome sequences of 5,156,544 bp for JCM 18905, 5,168,682 bp for JCM 19053, and 5,270,185 bp for JCM 18904, with 23×, 15×, and 23× redundancies and G+C contents of 44.9, 44.4, and 44.6%, respectively. A total of 5,001, 5,108, and 5,225 protein-coding genes and 77, 51, and 67 RNA-encoding sequences for JCM 18905, JCM 19053, and JCM 18904, respectively, were identified using the RAST server (4).

Phylogenetic analyses based on 16S rRNA gene sequence comparisons revealed that all the three strains were most closely related to the type strain of *Vibrio alginolyticus* (sequence identities, 98%, 99%, and 98%, for JCM 18905, JCM 19053, and JCM 18904, respectively) (5). The annotation of the genomes suggested that these three strains had versatile properties such as glutathione metabolism, secretion systems for effecter proteins and toxins, a quorum-sensing system, and production of signal molecules for inter-species communication. Glutathione plays an important role in conjugation and detoxication of xenobiotics. Preliminary comparative analyses showed that there were several genes present in the genomes of strains JCM 18905 and JCM 19053 (isolated from TTX-bearing organisms) but absent in the genomes of strain JCM 18904 and the type strain of *Vibrio alginolyticus*, NBRC 15630. These genes may be related to the TTX production. The genome information of these strains will be useful for studies of TTX production in bacteria.

### Nucleotide sequence accession numbers.

The genome sequences of *Vibrio* sp. strains JCM 18905, JCM 19053, and JCM 18904 have been deposited at DDBJ/EMBL/GenBank database under the accession no. BAYS01000001 to BAYS01000064, BAXK01000001 to BAXK01000106, and BAYR01000001 to BAYR01000070, respectively.
